# Novel Quinazolinone MJ-29 Triggers Endoplasmic Reticulum Stress and Intrinsic Apoptosis in Murine Leukemia WEHI-3 Cells and Inhibits Leukemic Mice

**DOI:** 10.1371/journal.pone.0036831

**Published:** 2012-05-25

**Authors:** Chi-Cheng Lu, Jai-Sing Yang, Jo-Hua Chiang, Mann-Jen Hour, Kuei-Li Lin, Jen-Jyh Lin, Wen-Wen Huang, Minoru Tsuzuki, Tsung-Han Lee, Jing-Gung Chung

**Affiliations:** 1 Department of Life Sciences, National Chung Hsing University, Taichung, Taiwan; 2 Department of Pharmacology, China Medical University, Taichung, Taiwan; 3 School of Pharmacy, China Medical University, Taichung, Taiwan; 4 Department of Radiation Oncology, Chi Mei Medical Center, Tainan, Taiwan; 5 Graduate Institute of Chinese Medicine, China Medical University, Taichung, Taiwan; 6 Division of Cardiology, China Medical University Hospital, Taichung, Taiwan; 7 Department of Biological Science and Technology, China Medical University, Taichung, Taiwan; 8 Department of Biochemistry, Nihon Pharmaceutical University, Saitama, Japan; 9 Tsuzuki Institute for Traditional Medicine, China Medical University, Taichung, Taiwan; 10 Department of Biotechnology, Asia University, Taichung, Taiwan; Emory University, United States of America

## Abstract

The present study was to explore the biological responses of the newly compound, MJ-29 in murine myelomonocytic leukemia WEHI-3 cells *in vitro* and *in vivo* fates. We focused on the *in vitro* effects of MJ-29 on ER stress and mitochondria-dependent apoptotic death in WEHI-3 cells, and to hypothesize that MJ-29 might fully impair the orthotopic leukemic mice. Our results indicated that a concentration-dependent decrease of cell viability was shown in MJ-29-treated cells. DNA content was examined utilizing flow cytometry, whereas apoptotic populations were determined using annexin V/PI, DAPI staining and TUNEL assay. Increasing vital factors of mitochondrial dysfunction by MJ-29 were further investigated. Thus, MJ-29-provaked apoptosis of WEHI-3 cells is mediated through the intrinsic pathway. Importantly, intracellular Ca^2+^ release and ER stress-associated signaling also contributed to MJ-29-triggered cell apoptosis. We found that MJ-29 stimulated the protein levels of calpain 1, CHOP and p-eIF2α pathways in WEHI-3 cells. In *in vivo* experiments, intraperitoneal administration of MJ-29 significantly improved the total survival rate, enhanced body weight and attenuated enlarged spleen and liver tissues in leukemic mice. The infiltration of immature myeloblastic cells into splenic red pulp was reduced in MJ-29-treated leukemic mice. Moreover, MJ-29 increased the differentiations of T and B cells but decreased that of macrophages and monocytes. Additionally, MJ-29-stimulated immune responses might be involved in anti-leukemic activity *in vivo*. Based on these observations, MJ-29 suppresses WEHI-3 cells *in vitro* and *in vivo*, and it is proposed that this potent and selective agent could be a new chemotherapeutic candidate for anti-leukemia in the future.

## Introduction

Leukemia, a group of hematologic malignancies disorder in leukocytes, is characterized by the uncontrolled proliferation and blocked in differentiation of hematopoietic cells [1], [2] and subdivided into acute and chronic forms [3]. Among most human leukemias, they exhibit the blockage of differentiation, enhancement of viability and dysregulation of cell cycle control that is necessary for occurrences of malignant transformation [4]. In the United States, leukemia is the largest number of cases of childhood cancer (approximately 2,000 cases per year) [5]. In Taiwan, a 2010 report from the Department of Health, R.O.C. (Taiwan) indicated that approximately 4.2 per 100,000 individuals die annually from leukemia [6]. The current clinical trials for leukemia include the pharmaceutical medications, debilitating radiation, and a bone marrow transplant therapy but these strategies have not proven to be satisfied. Hence, new targets for treating leukemia are necessary and the best functions for agents are carried out through promoting differentiation or trigging apoptotic death in leukemia cells [7], [8]. Apoptosis, a process of programmed cell death type I, is a major method of anticancer properties to eliminate cancer cells [9]. The mitochondrial depolarization and activations of caspase family proteases are the central steps when the development of apoptosis [10], and their associated signaling pathways include intrinsic (mitochondria-dependent) and ER stress (unfolded protein response) signals [11], [12].

Numerous phytochemicals are known to present in many herbal based dietary supplements or herbal medicines, which might be effective in clinical applications and used as cancer suppressors; these molecules are invaluable contributions of nature [13], [14]. It has been reported that the microtubule-targeting agents (MTAs) are one of the most effective drugs in leukemia [15] but they exert side effects and high toxicity on normal tissues after treating to patients [16], [17]. However, the current effective chemotherapeutic agents, such as taxanes and vinca have limitations and are not satisfying leukemic therapies because of toxic side effects and drug resistance [18]. Seeking novel agents for chemotherapy-induced apoptotic death is not only becoming more important and essential but has received increasing attention in the leukemia patients [19].

The previous reports have shown that alkaloids with 4-quinazolinone nuclei possess various biological functions (anti-inflammatory, anti-bacterial and anti-malarial) and antitumor effects [20], [21]. In our cooperative laboratory, a series of 2-phenyl 6-pyrrolidinyl-4-quinazolinone derivatives have been designed and synthesized, and which are found to have anti-mitotic functions and anticancer activities in many types of tumor cell lines, including colorectal, lung, ovarian, oral, prostate and breast cancer as well as glioblastoma, osteosarcoma, melanoma and leukemia [20], [22], [23]. This novel agent, 6-pyrrolidinyl-2-(2-hydroxyphenyl)-4-quinazolinone (MJ-29) exhibits the most potent cytotoxicity against leukemia cell lines [20]. Our earlier study also indicated that MJ-29 inhibited tubulin polymerization, induced mitotic arrest and provoked apoptosis in a human leukemic monocyte lymphoma cell line (U937), and that attenuated U937 xenograts tumor growth *in vivo* [21]. Until now, the anticancer actions of the newly quinazolinone compound, MJ-29 on murine leukemia cells *in vitro* and *in vivo* are not yet completely understood. The objectives of this study are to verify the hypothesis that MJ-29 might influence the murine myelomonocytic leukemia cell line (WEHI-3), as was the underlying mechanisms by MJ-29 might induce ER stress and mitochondria-mediated apoptosis, and further evaluate anti-leukemic activity in orthotopic model of leukemic mice.

## Results

### MJ-29 induces cytotoxicity and morphological changes in murine leukemia WEHI-3 cells

**Figure 1:**
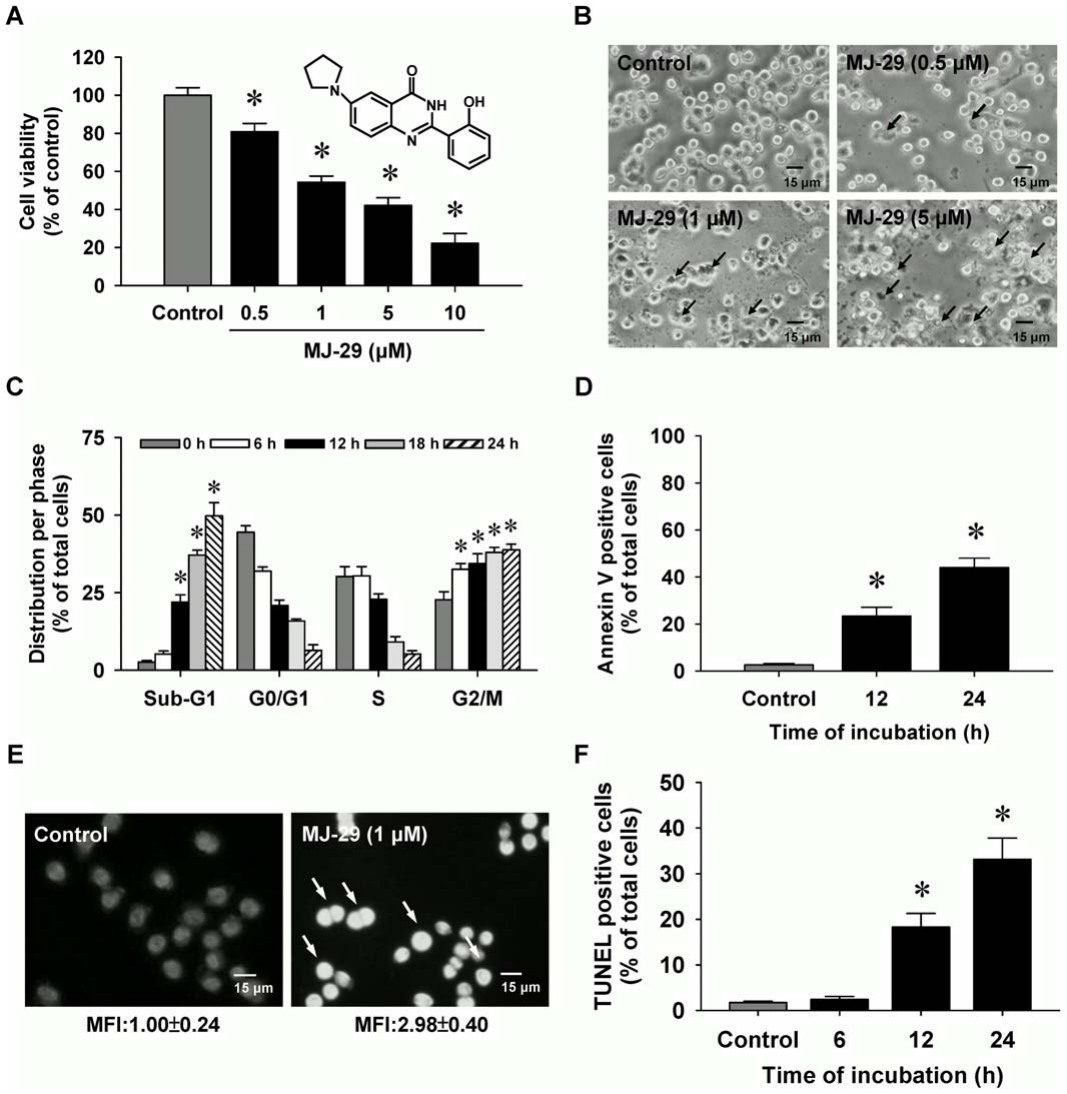
MJ-29 decreases the viability and induces apoptotic death in WEHI-3 cells. Cells were treated with or without 0.5, 1, 5 or 10 µM of MJ-29 for 24 h and exposed to 1 µM of MJ-29 for indicated durations. (A) Cell viability was determined by a PI exclusion method and analyzed by flow cytometry (B) before the investigations for the cells' morphological changes were observed (↑ reveals the shrinkage and rounding of apoptotic cells) and photographed under phase-contract microscopy (scale bar, 15 µm). (C) Cells with G2/M phase and hypodiploid DNA contents (%) represent the fractions undergoing apoptotic DNA degradation. (D) Quantification of annexin V positive cells was measured using Annexin V FITC/PI kit and examined by flow cytometry. (E) DAPI staining and a fluorescent microscope were used to analyze chromatin condensation (a catachrestic of apoptosis) in MJ-29-treated cells. The arrow bar (↑) shows chromatin condensations in apoptotic cells due to their higher fluorescent intensity compared to the vehicle control group (scale bar, 15 µm). MFI of DAPI was measured and quantified. (F) TUNEL positive cells were determined and quantified by flow cytometry. Each assay described in the “Materials and Methods”. Each point is a mean ± S.D. of three independent experiments. *p<0.05 is significantly different compared with the 0.1% (v/v) DMSO-treated vehicle control by Tukey's HSD test.

Cells were exposed to MJ-29 at the concentrations of 0, 0.5, 1, 5 or 10 µM for a 24-h treatment. The potential cytotoxic effects of MJ-29 on WEHI-3 cells were investigated for cell viability by a propidium iodide (PI) exclusion method and using flow cytometric analysis. Results in Figure 1A showed that MJ-29 decreased the percentage of viable cells in WEHI-3 cells in a concentration-dependent response. We also confirmed that MJ-29 concentration-dependently reduced the cell viability by MTT assay (Figure S1A and Method S1). Figure 1B indicates that WEHI-3 cells were morphologically-altered by MJ-29 treatment (such as cell rounding and shrinkage) and these effects were concentration-dependent. The half-maximal effective concentration (EC_50_) value of MJ-29 for 24-h exposure was 1.03±0.29 µM after the non-linear dose-response regression curve was fitted by SigmaPlot 10 (Systat Software, Inc. San Jose, CA, USA) [24], [25]. Therefore, MJ-29 at the concentration of 1 µM was selected for further experiments in this study. Importantly, our earlier study has reported that MJ-29 exhibited less toxicity in normal cells, including peripheral blood mononuclear cells (PBMC) and human umbilical vein endothelial cells (HUVECs) in comparison to that in the higher sensitive WEHI-3 cells [21].

### MJ-29 triggers G2/M phase arrest and provokes apoptosis in WEHI-3 cells

To verify MJ-29-induced cell death through G2/M phase arrest and apoptotic death, cells were treated with MJ-29 before analyses with sub-G1 population (apoptosis), Annexin V FITC/PI kit, 4′,6-diamidino-2-phenylindole (DAPI) staining and terminal DNA transferase-mediated dUTP nick end labeling (TUNEL) assays. The results revealed that MJ-29 induced G2/M phase arrest from 23.31% to 77.89%, and it increased the sub-G1 group from 2.63% to 49.7% in WEHI-3 cells (Figure 1C and Figure S1B). Figure 1D and Figure S1C show that the apoptotic cells (annexin V positive cells) increased from 2.0% to 39.5% within 24 h between the control sample and MJ-29-treated cells. Also, these effects are to undergo a time-dependent association in MJ-29-treated WEHI-3 cells. Moreover, MJ-29 caused chromatin condensation (a characteristic of apoptosis) in WEHI-3 cells as shown by an increase in mean fluorescence intensity (MFI) (Figure 1E). As demonstrated in Figure 1F, MJ-29 exposure for 0, 6, 12 and 24 h time-dependently stimulated the appearance of TUNEL positive cells, causing that the DNA fragmentation occurred in WEHI-3 cells.

### MJ-29 stimulates mitochondrial dysfunction in WEHI-3 cells

**Figure 2:**
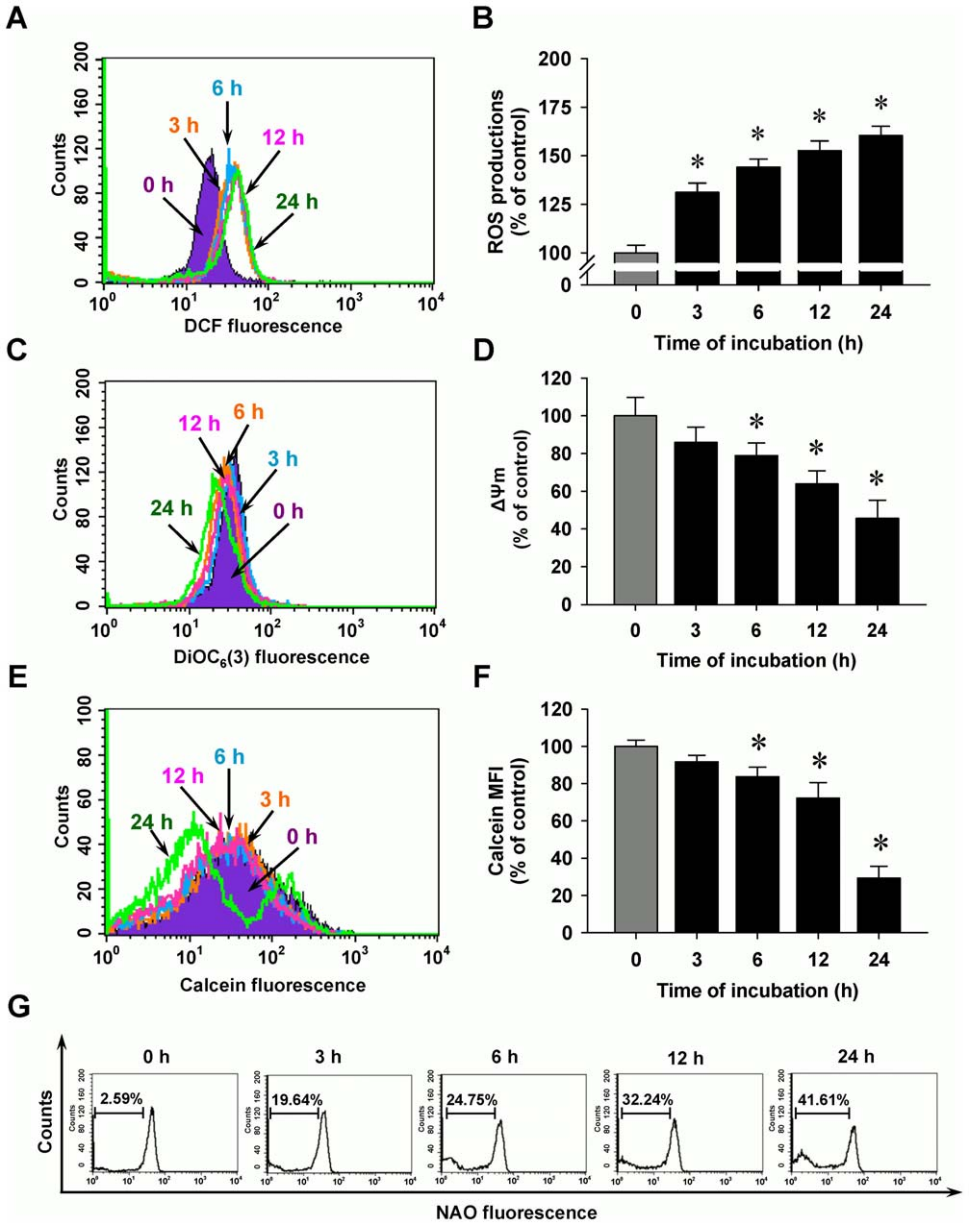
MJ-29 enhances ROS productions and promotes mitochondrial dysfunctions in WEHI-3 cell apoptosis. Cells were incubated with 1 µM of MJ-29 for indicated periods of time (3, 6, 12 and 24 h) and then were harvested for examining the (A) ROS productions, (C) the level of ΔΨm and (E) MPT pores opening as described in the “Materials and Methods”. (B, D, F) Quantification of these responses was displayed and determined by BD CellQuest Pro software. The results are shown as means ± S.D. (n = 3) and significant different (*p<0.05) was considered when compared to the 0.1% (v/v) DMSO vehicle control (0 h) by Tukey's HSD test. (G) NAO fluorescence by flow cytometry was performed for cardiolipin oxidation to evaluate a left shift. Representative images are taken from three independent experiments.

To evaluate whether MJ-29 influences crucial factors in mitochondria and investigate the roles of mitochondria-regulated death pathways, our results showed that MJ-29 depolarized the level of mitochondrial membrane potential (ΔΨm) (Figures 2C and D), promoted the opening of the mitochondrial permeability transition (MPT) pores (Figures 2E and F) and triggered level of cardiolipin oxidation (Figure 2G) in WEHI-3 cells. The responses occurred in a time-course effect. These data indicated that treatment of WEHI-3 cells by MJ-29 which induced the cell apoptosis, disrupted the ΔΨm and provoked mitochondrial depolarization. It is reported that mitochondrial dysfunction might result from oxidative stress, leading to cardiolipin oxidation [26], [27]. We further investigated that if oxidative stress influences the upstream of mitochondrial dysfunction, and our findings demonstrated that MJ-29 increased ROS levels up to 24-h treatment in WEHI-3 cells as shown in Figures 2A and B.

### MJ-29 triggers cell death in WEHI-3 cells through the intrinsic apoptotic pathway

**Figure 3:**
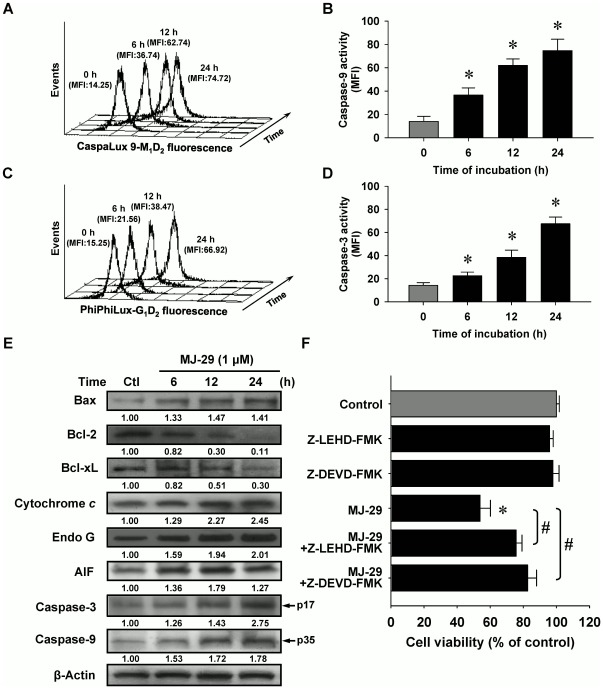
MJ-29 triggers apoptotic death in WEHI-3 cells through the intrinsic signaling pathway. Cells were pretreated in the presence or absence of the specific inhibitors of caspase-9 (Z-LEHD-FMK) and caspase-3 (Z-DEVD-FMK) for 2 h and then were exposed to 1 µM of MJ-29 for 6, 12 or 24 h. Flow cytometry analysis was used to determine caspase-9 (A) and caspase-3 (C) activity and respective profiles were shown using CellQuest Pro software (B and D). MFI indicates mean fluorescence intensity. (E) Cell extracts were prepared to determine by Western blotting analysis for protein levels of Bax, Bcl-2, Bcl-xL, cytochrome *c*, Endo G, AIF, cleaved caspase-3 and cleaved caspase-9. β-Actin was used as a loading control. (F) Pretreatments with Z-LEHD-FMK and Z-DEVD-FMK followed exposure to MJ-29 were determined by a PI exclusion method as described in the “Materials and Methods”. Columns, mean (n = 3); bars, SD. *, *p*<0.05, is significantly different compared with 0.1% (v/v) DMSO vehicle control and #, *p*<0.05 significantly greater than values obtained for cells treated with MJ-29 alone by Tukey's HSD test.

**Figure 4:**
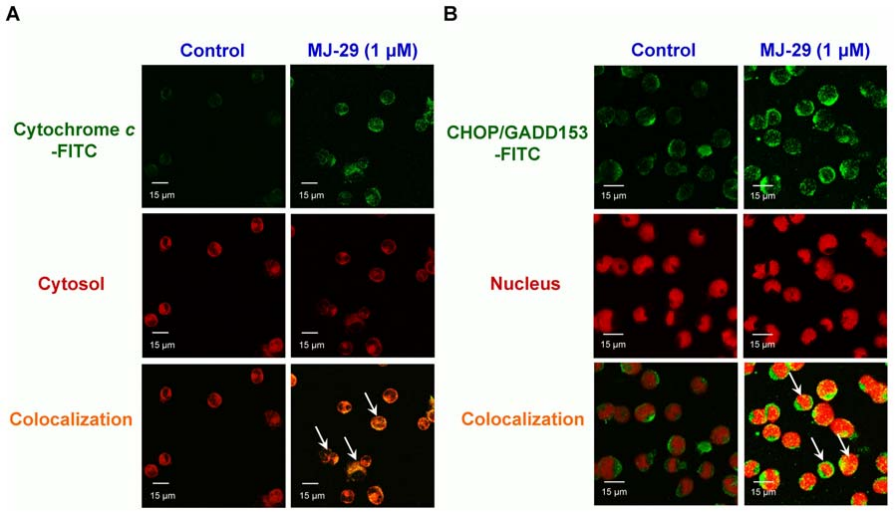
MJ-29 stimulates the translocations of cytochrome *c* and CHOP/GADD153 levels to cytosol or nucleus in WEHI-3 cells. Cells (5×10^4^ cells/well) plated on 4-well chamber slides were incubated in the presence or absence with 1 µM of MJ-29 for 24 h before being stained by the antibodies against (A) cytychrome *c* and (B) CHOP/GADD153, and then FITC-labeled secondary antibodies were applied (green fluorescence). Cytosol and nucleus were counterstained with CellTracker Red CMTPX and PI, respectively (red color). 0.1% (v/v) DMSO alone served as a vehicle control. The images were visualized under a confocal microscope as described in the “Materials and Methods”. Scale bar, 15 µm. Data are representative of three independent experiments that yielded similar results.

Our data in Figure 3 indicated that MJ-29-induced apoptosis was mediated by stimulating caspase-9 (Figures 3A and B) and caspase-3 (Figures 3C and D) activities in a time-dependent effect. Figure 3E indicates that MJ-29 up-regulated the protein levels of Bax, cytochrome *c*, Endo G, AIF, cleaved caspase-3 (p17) and cleaved caspase-9 (p35) but it down-regulated that of Bcl-2 and Bcl-xL (Figure 3C) in WEHI-3 cells, reflecting the apoptotic states of WEHI-3 cells. Additionally, the trafficking of cytochrome *c* from mitochondria to cytosol was stimulated in MJ-29-treated WEHI-3 cells as illustrated in Figure 4A. To confirm if MJ-29-induced apoptosis is involved in caspase-9 and caspase-3-mediated mitochondria-dependent signaling, cells were individually pretreated with specific caspase-9 (Z-LEHD-FMK) and caspase-3 (Z-DEVD-FMK) inhibitors before 1 µM of MJ-29 for 24 h. Results in Figure 3F showed that both specific inhibitors substantially reduced the effects of viability (cell death) on WEHI-3 cells, resuljting in more viable cells when compared to the MJ-29-treated alone sample. The current evidence suggests that the activations of caspase-9 and caspase-3 might fully contribute to MJ-29-triggered apoptotic death in WEHI-3 cells. Therefore, MJ-29-enhanced apoptosis in murine leukemia WEHI-3 cells was carried out mainly by the activations of caspase-9 and caspase-3-mediated mitochondrial signaling pathways.

### Intracellular Ca^2+^ release and unfolded protein response are associated with the induction of apoptosis in MJ-29-treated WEHI-3 cells

**Figure 5:**
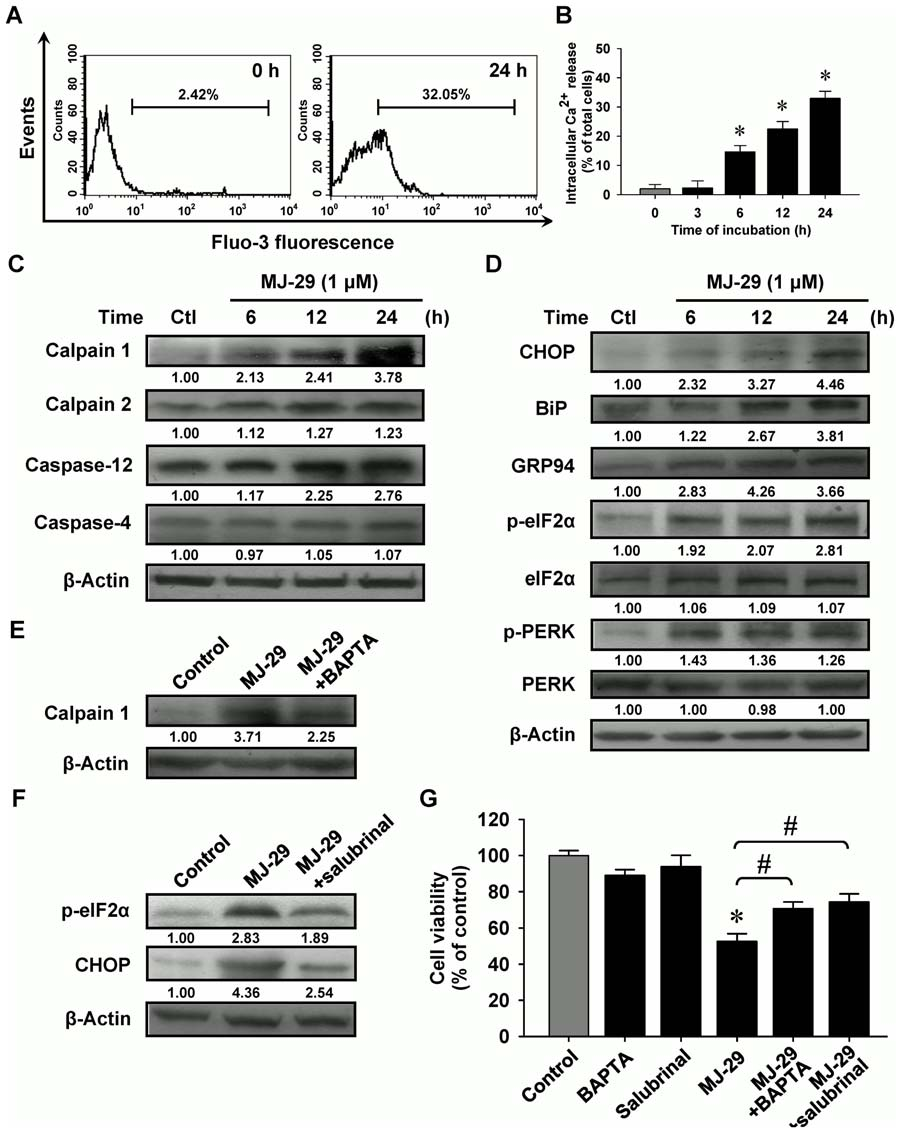
MJ-29 provokes intracellular Ca^2+^ release and unfolded protein response-related hallmark protein expressions in WEHI-3 cells. (A) Cells were incubated with or without 1 µM of MJ-29 at the indicated intervals of time for measuring the fluorescence intensity of Fluo-3 by flow cytometry, and (B) data were analyzed using BD CellQuest Pro software. Cells were harvested and the indicated proteins levels [(C): calpain 1, calpain 2, caspase-12 and caspase-4; (D): CHOP, BiP, GRP97, p-eIF2α, eIF2α, p-PERK and PERK] were subjected to Western blotting. To confirm the effects of BAPTA or salubrinal on MJ-29-induced cell death, cells were individually pretreated with or without 5 µM BAPTA or 10 µM salubrinal for 2 h and further treated with 1 µM of MJ-29 following a 24-h exposure. Whole-cell protein lysates were prepared and performed for detecting the protein levels of (E) calpain 1 and (F) p-eIF2α by immunoblotting. β-Actin served for loading control. (G) Cell viability was determined by a PI exclusion method and flow cytometry. Data are expressed as overall means ± S.D. from three independent experiments. Statistical significance was determined by Tukey's HSD test. *, *p*<0.05, shows significant difference compared with 0.1% (v/v) DMSO vehicle control; #, *p*<0.05, is significantly different compared to MJ-29 treatment alone.

To elucidate the upstream possible signaling pathways of MJ-29-induced cell death of WEHI-3 cells, we tested whether intracellular Ca^2+^ induction contributes to MJ-29-activated apoptotic signaling. Figures 5A and B display that cells were incubated with 1 µM of MJ-29 for 3 h to 24 h and it is found that MJ-29 significantly increased cytosolic Ca^2+^ level in WEHI-3 cells. Many reports stated that activation of calpain, a member of calcium-dependent proteases, is implicated with ER stress and perturbations intercellular Ca^2+^ release in mammalian cells [28], [29]. As a result shown in Figure 5B, the expressions of these ER stress-related protein levels modulated by intercellular Ca^2+^ and caspase signals, including calpain 1, calpain 2 and caspase-12 in WEHI-3 cells were time-dependently induced after MJ-29 treatment. However, the protein level of casepase-4 was not dramatically increased in MJ-29-treated WEHI-3 cells (Figure 5C).

To determine whether MJ-29 could induce ER stress, we investigated several vital hallmarks of UPR, including C/EBP homologous protein (CHOP), immunoglobulin heavy chain binding protein (BiP), glucose-regulated protein 94 (GRP94), alpha subunit of eukaryotic initiation factor 2 (eIF2α) and PRK (RNA-dependent protein kinase)-like ER kinase (PERK) proteins levels. Results in Figure 5D demonstrated that the increased expressions of CHOP, BiP and GRP94 at 6 to 24-h exposure in WEHI-3 cells after MJ-29 exposure. Also, treatment with MJ-29 promoted the translocation of CHOP/GADD153 level to nucleus in WEHI-3 cells (Figure 4B). Additionally, we examined whether MJ-29 affects the phosphorylation of eIF2α and PERK, which are the ER stress-associated important protein levels. Results revealed that MJ-29 induced an increase in the protein levels of p-eIF2α and p-PERK during the time period of 6–24 h in WEHI-3 cells (Figure 5D). Moreover, we determined if intracellular Ca^2+^ and UPR-related signals are involved in modulating the MJ-29-induced ER stress through activating calpain 1 and CHOP/eIF2α signaling pathways. We compared the effects of MJ-29-treated WEHI-3 cells on the levels of calpain 1, p-eIF2α and CHOP in the absence and presence of BAPTA or salubrinal, respectively. Results in Figure 5E showed that BAPTA (a Ca^2+^ chelator) that is effective at suppressing MJ-29-induced calpain 1 protein level compared with only treated WEHI-3 cells. Alternatively, pretreatment with salubrinal (an eIF2α dephosphorylation inhibitor) resulted in the decreased MJ-29-activated the levels of p-eIF2α and CHOP in WEHI-3 cells (Figure 5F). As seen in Figure 5G, reduction of cell viability in WEHI-3 cells by MJ-29 was significantly decreased not only by BAPTA but also by salubrinal in comparison to MJ-29-treated only cells. Overall, data in Figure 5 clearly demonstrated that stimulation of unfolded protein response might be responsible for increased intracellular Ca^2+^ release as well as calpain 1 and CHOP/eIF2α signaling pathways in MJ-29-treated WEHI-3 cells.

### MJ-29 prolongs the survival rate and enhances the body weight of WEHI-3 leukemic BALB/c mice

**Figure 6:**
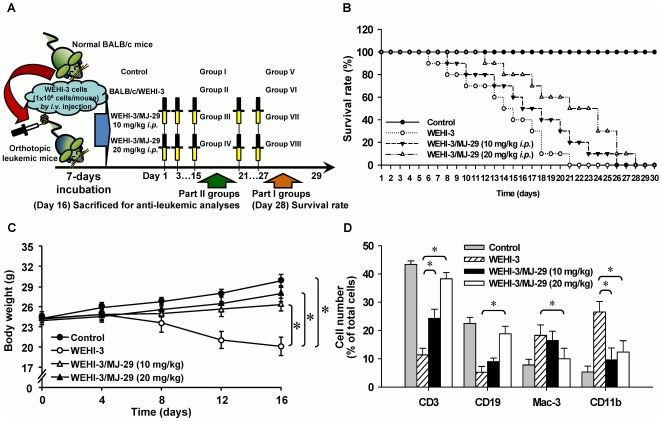
Anti-leukemic activity of MJ-29-impaired WEHI-3 leukemic mice *in vivo*. (A) The experimental design and protocol of orthotopic leukemic mice model. Efficacy of intraperitoneal (*i.p.*) treatment with MJ-29 was investigated the survival rate and anti-leukemic responses on leukemic mice [BALB/c mice after intraperitoneal (*i.v.*) with WEHI-3 cells]. (B) Whole survival rate of leukemic mice was counted after MJ-29 exposure for 28-days administration. The animals were given intraperitoneally and anti-leukemic activity was determined as the survival rate by Kaplan-Meier estimator every day in all groups. There is a significant overall survival difference in comparison to the leukemic mice groups in the presence and absence of MJ-29 exposure versus control mice. (C) Mice were intravenously injected with WEHI-3 cells (1×10^6^ cells/100 µl per mouse) in PBS, and then treated with or without MJ-29 (10 and 20 mg/kg) by intraperitoneal injection for 16 days. During the treatment, each animal was measured the body weight once every four days for 16-days intervals as described in the “Materials and Methods”. (D) Whole blood was collected from individual mice and leukocytes were analyzed the with specific cell surface markers by flow cytometry. The CD markers include CD3 for T lymphocytes, CD19 for B cells, Mac-3 for macrophages and CD11b for monocytes. The results are expressed as means ± S.D. and samples were obtained from at least five mice per group, and * *p*<0.05 is found significantly different by Tukey's HSD test when compared with the leukemic (only injection with WEHI-3 cells) and experimental (WEHI-3 cells injected before intraperitoneal treatment with MJ-29 at 10 and 20 mg/kg) mice groups.

We have previously established the leukemic animal model to test the effects of MJ-29 on the survival rate in WEHI-3 leukemic mice [30], [31]. Our experimental design and protocol of the different treatment groups are shown in Figure 6A. We intraperitoneally administrated with MJ-29 (10 and 20 mg/kg, respectively) in mice after being inoculated with WEHI-3 cells. As displayed in Figure 6B, MJ-29 significantly prolonged the survival time of mice with median survival time (MST) by 23.8% and 33.3% (median, 21 days for control mice versus 26 and 28 days for mice treated with MJ-29 at 10 and 20 mg/kg, respectively) using Kaplan-Meier estimator and there was 5- and 7-days prolongations in average life span of leukemic mice until 28 days. Furthermore, MJ-29 (10 and 20 mg/kg) promoted body weight in leukemic mice (WEHI-3/BALB/c: 20.32±1.82 g; WEHI-3/BALB/c/MJ-29 10 mg/kg: 25.63±0.76 g; WEHI-3/BALB/c/MJ-29 20 mg/kg: 26.71±1.24 g) for 16-days exposure with intraperitoneal treatment (Figure 6C).

### MJ-29 alters the CD markers of leukocytes from PBMC in leukemic mice

The normal and leukemic mice were treated with or without MJ-29 (10 and 20 mg/kg) for 16 days. Blood samples were collected from individual animals of each group, and then leukocytes were stained with anti-CD3 for T cells, anti-CD19 for B cells, anti-Mac-3 for macrophages and anti-CD11b for monocytes, respectively. Results shown in Figure 6D and Figure S3 indicated that MJ-29 induced the levels of CD3 (10 mg/kg: an increase of 12.3%; 20 mg/kg: an increase of 28.3%) and CD19 (10 mg/kg: an increase of 3.6%; 20 mg/kg: an increase of 14.3%). Based on these observations, both of the effects were dose-dependent in leukemic mice. However, MJ-29 reduced the levels of Mac-3 (10 mg/kg: a decrease of 2.5%; 20 mg/kg: a decrease of 9.0%) and CD11b (10 mg/kg: a decrease of 18.1%; 20 mg/kg: a decrease of 14.4%) in comparison to the only WEHI-3 cells-injected mice group. Therefore, intraperitoneal administration with MJ-29 to leukemic mice altered the specific surface markers from PBMC *in vivo*.

### MJ-29 affects the weights of spleen and liver tissues as well as splenic histopathology in leukemic mice

**Figure 7:**
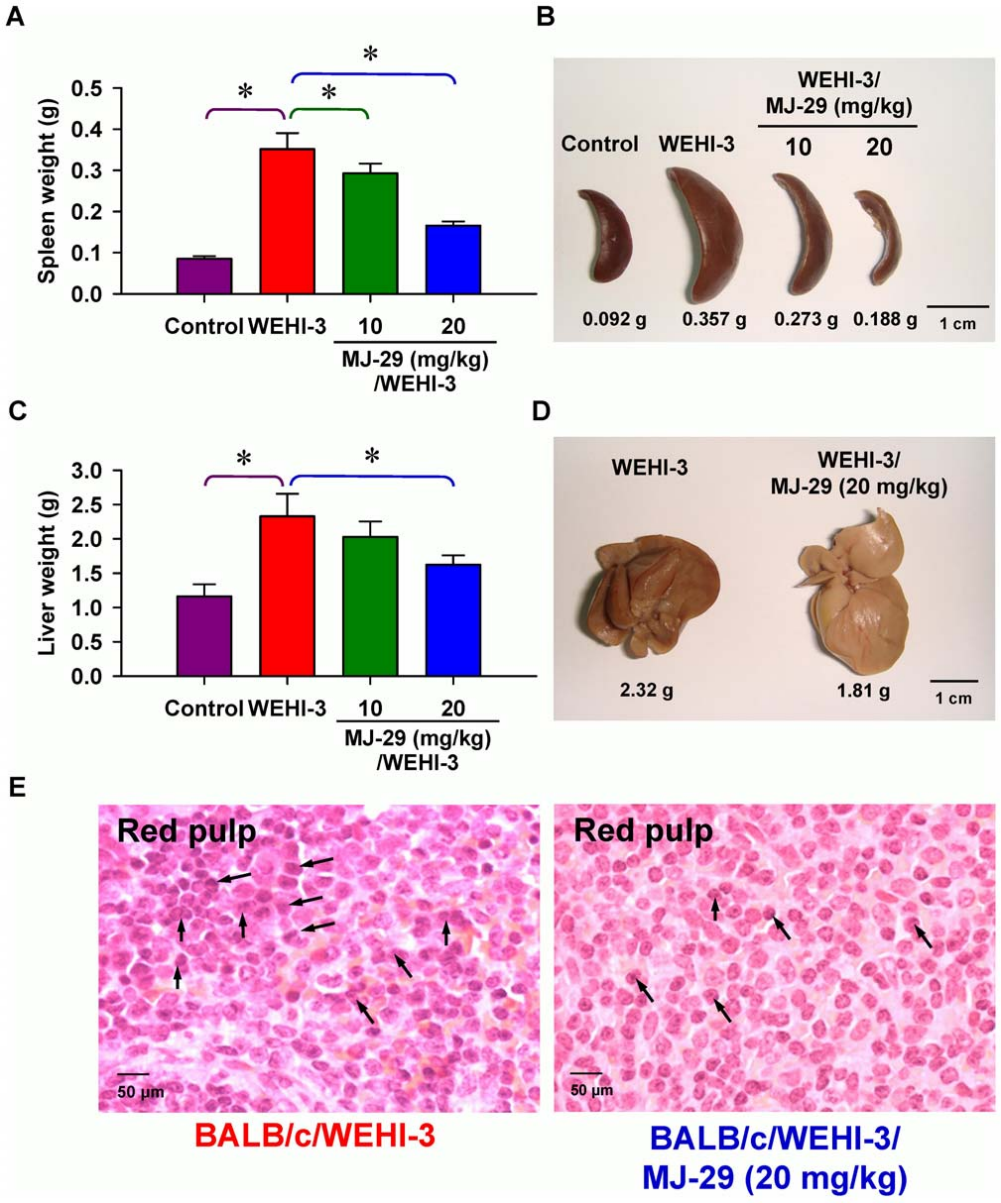
Effects of the weights in spleen and liver as well as histopathological examination of spleen tissues on MJ-29-treated leukemic mice. Animals were intravenously injected with WEHI-3 cells (1×10^6^ cells/100 µl per mouse) in PBS, and then treated intraperitoneally with MJ-29 (10 and 20 mg/kg alternate day for 8 times). Weights and representative images of spleen (A and B) and liver (C and D) tissues from leukemic mice were determined and measured individually. Each point is mean ± S.D. (at least five samples). * *p*<0.05 indicates significant difference by Tukey's HSD test between the WEHI-3 leukemic mice and experimental (normal or intraperitoneal treatment with MJ-29 at 10 and 20 mg/kg, respectively) groups. (E) Dissected leukemic mice and hematoxylin-eosin stain for the paraffin sections of spleens from MJ-29-treated and un-treated leukemic mice as described in the “Materials and Methods”. Arrows (↑) shows infiltration of immature myeloblastic cells (leukemia cells) into red pulp of the spleen. R, red pulp. The data are performed with representative experiment in triplicate and three independent experiments with similar results.

Each spleen or liver tissue isolated from the normal and leukemic mice groups was weighed. As can be seen in Figures 7A and B, both doses (10 and 20 mg/kg) of MJ-29 treatment significantly decreased the weights of spleen (the decreases of 16.7% and 52.8%, respectively) between WEHI-3 leukemic mice with or without MJ-29 intraperitoneal administration. Also, MJ-29 at 20 mg/kg significantly reduced the weight of liver tissues (a decrease of 30.3%) in comparison to only WEHI-3 cells-injected mice *in vivo* (Figures 7C and D). Moreover, the representative results of histopathological examination are presented in Figure 7E, which indicates that the infiltration of immature myeloblastic cells into splenic red pulp (R) in spleen section was eliminated in MJ-29 (20 mg/kg)-treated leukemic mice (Figure 7E, right panel) when compared to untreated-mice after intravenous injection with WEHI-3 cells. Little differences were shown that marked expansion in the R area rather than that in the white pulp. The neoplastic cells contained large irregular nuclei accompanied with clumped chromatin and prominent nucleoli versus abundantly clear and light eosinophilic cytoplasm. Also, the number of megakaryocytes increased in MJ-29-treated leukemic animals (Figure 7E).

### MJ-29 enhances the phagocytosis by macrophages as well as the T- and B-cell proliferations and NK cell cytotoxicity in leukemic mice

**Figure 8:**
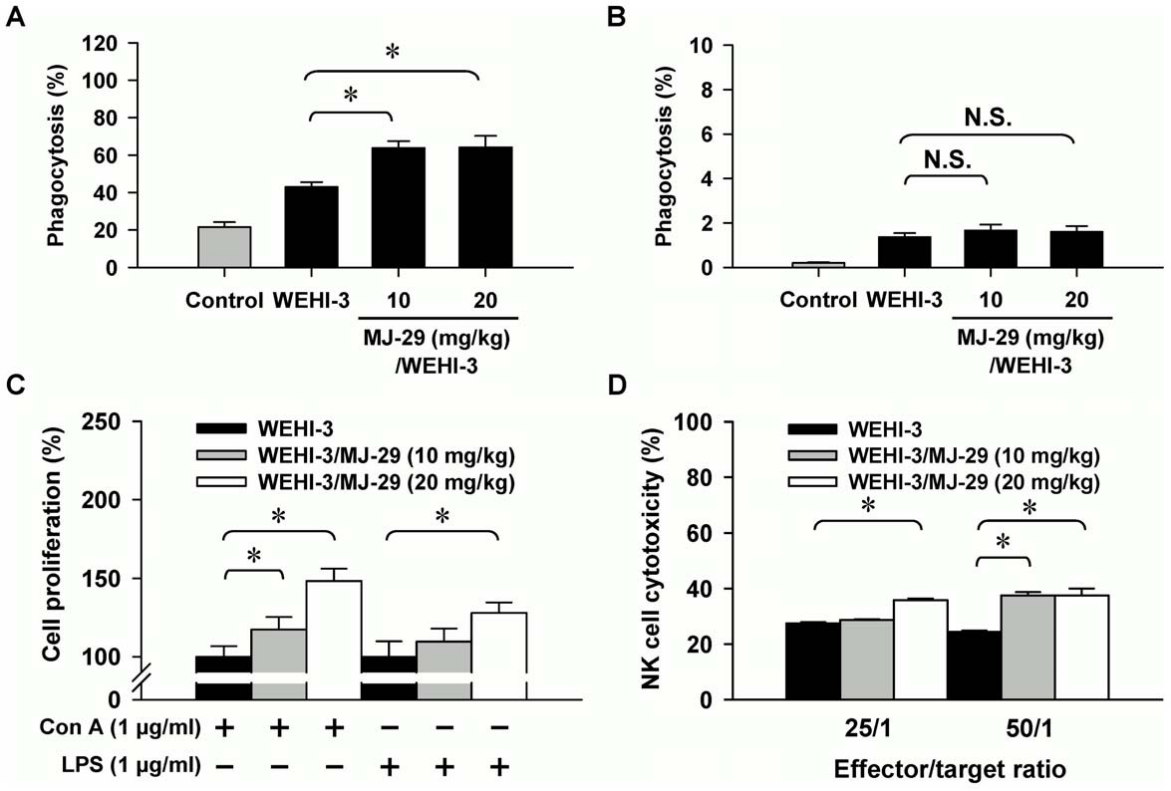
MJ-29 alters phagocytosis by macrophages from PBMC as well as T- and B-cell proliferations and NK cell cytotoxicity from splenocytes in leukemic mice. Mice were intravenously injected with WEHI-3 cells (1×10^6^ cells/100 µl per mouse) and intraperitoneally treated with MJ-29 (10 and 20 mg/kg, respectively) for 16 days. Macrophages were isolated from (A) PBMC and (B) peritoneal cavity of each group. The percentage of phagocytic leukocytes that was ingesting FITC-*E. coli*, and the samples were determined using flow cytometry. Splenocytes were isolated from each mouse of groups for (C) T- and B-cell proliferation examinations and (D) NK cell cytotoxicity as described in the “Materials and Methods”. Each point is mean ± S.D. for at least five samples per group. * *p*<0.05 is considered significant by Tukey's HSD test when compared with the untreated WEHI-3 leukemic mice group.

To explore if MJ-29 affects phagocytosis, the leukocytes from MJ-29-treated or untreated mice were isolated and phagocytic activity by macrophages was determined which can be seen in Figures 8A and B. Our results revealed that MJ-29 (10 and 20 mg/kg, respectively) enhanced phagocytosis from PBMC (10 mg/kg: 18.4%; 20 mg/kg/day: 20.6%) (Figure 8A) but it did not significantly influence that from peritoneal cavity (Figure 8B) by comparison to untreated leukemic mice. Thereafter, we further investigated that whether MJ-29 promotes T- and B-cell proliferations from splenocytes in leukemic mice. Our results in Figure 8C indicated that both doses of MJ-29 increased T-cell proliferation after concanavalin A (Con A) stimulation but it only promoted B-cell proliferation by co-treatment with lipopolysaccharide (LPS) when leukemic mice after 20 mg/kg MJ-29 exposure. Furthermore, splenocytes from MJ-29-treated or untreated mice were isolated and determined NK cell cytotoxicity *in vivo*. MJ-29 at 20 mg/kg was effective at both target ratios of 50/1 and 25/1 but only MJ-29 at 10 mg/kg-tested dose showed a significant effect at the ratio of 25/1 when compared to the untreated control leukemic mice (Figure 8D).

## Discussion

Numerous reports have described that novel agents for therapies based on targeting on interference of microtubule assembling provided a promising insight toward a cure for leukemia [14], [32], [33]. Recently, we have synthesized the 2-phenyl-4-quinazolinone analogs which are found to have anti-mitotic functions and bioactivities in many types of cancer cell lines, including human leukemia cells [20], [22], [23]. The previous study has shown that MJ-29 possessed the suppression of the proliferation and induction of apoptosis in human leukemia U937 cells [21]. Also, the *in vivo* study showed that MJ-29 inhibited tumor growth of xenografts in nude mice. In the current study, our results presented here clearly demonstrated that MJ-29 could modulate antiproliferative effects and trigger apoptosis caused by ER stress and intrinsic pathways in WEHI-3 cells. Furthermore, MJ-29 could prolong the survival rate in leukemic mice that might be involved in levels of specific cell surface markers and alterations of immunemodulation *in vivo*. Therefore, this study strongly suggests that the newly quinazolinone compound, MJ-29 triggers cell apoptosis in murine leukemia WEHI-3 cells *in vitro* and suppresses orthotopic leukemic mice *in vivo*.

We therefore examined the cytotoxicity of MJ-29 in WEHI-3 cells and investigated its molecular mechanisms *in vitro*. Our data revealed that MJ-29 at the concentrations of 0.5–10 µM had the concentration-dependent cytotoxic effect (Figure 1A and Figure S1A) and induced morphological changes, such as cell shrinkage and rounding (Figure 1B) on WEHI-3 leukemia cells. Additionally, our earlier study indicated that MJ-29 exerted a less cytotoxicity in normal cells (PBMC and HUVECs) and EC_50_ value of both is more than 10 µM of MJ-29 for a 24-h treatment [21]. It is notable that MJ-29 could represent a promising agent for anti-leukemia, and there are more safety properties, fewer side effects and selectivity of actions on normal cells as compared to that of paclitaxel and vincristine based on the critical factors, such as doubling time and EC_50_ value of PBMC and HUVECs [21].

It has been generally accepted that induction of apoptosis is a best strategy or option by many chemotherapeutic agents for antitumor and anti-leukemia treatments [18], [34], and the apoptotic characteristics include oligonucleosomal DNA fragmentation, membrane blebbing, etc [27]. In the present study, we examined antitumor effects of MJ-29 on WEHI-3 cells by investigating mitochondrial dysfunction-associated apoptotic signaling pathway. The apoptotic WEHI-3 cells were determined with four different assays, containing DNA content analysis, annexin V population assessment, DAPI staining and TUNEL assay. Results showed that MJ-29 treatment promoted G2/M phase arrest and increased the number of cells in the sub-G1 peak (apoptotic cells) (Figure 1C). Furthermore, MJ-29-triggered apoptotic populations were confirmed by annexin V/PI assay, DAPI staining and TUNEL assay. Our data demonstrated that MJ-29-increased the early (annexin V positive and PI negative) and late (annexin V positive and PI positive) apoptotic death can be distinguished in WEHI-3 cells (Figure 1D and Figure S1C), suggesting that the phospholipid phosphatidylserine (PS) is trafficking the outer leaflet since cell apoptosis [35]. Also, we found that MJ-29 caused chromatin condensation (a characteristic of cell apoptosis) (Figure 1E) and DNA fragmentations (Figure 1F) in WEHI-3 cells. These findings provided vital insights showing that MJ-29-induced cytotoxicity is mediated by inducing apoptotic death in WEHI-3 cells. This is also in agreement with our previous study addressing MJ-29-induced apoptosis in human leukemia cells, which was involved in the dysfunction of mitotic spindles, induction of mitotic arrest and cell apoptosis [21]. Importantly, MJ-29-stimulated CDK1 activation appears to be acting the phosphorylation of Bcl-2 (Ser70), resulting in promotion of the intrinsic apoptotic signaling in human leukemia U937 cells [21].

Mitochondria are one of primary key targets and ROS caused an increase of mitochondrial depolarization, leading to caspase cascades activations when tumor cell apoptosis [27], [36]. In this study, we displayed that MJ-29 increased ΔΨm dissipation (Figure 2D), MPT pores opening (Figure 2F), and activations of caspase-9 and caspase-3 (Figure 3), following inductions of mitochondria-mediated apoptosis in WEHI-3 cells. Also, the effects of cell death were significantly blocked after individual pretreatment with specific caspase-9 and caspase-3 inhibitors (Figure 3F) in WEHI-3 cells. Additionally, oxidative stress could promote a decrease the level of cardiolipin oxidation which is known to lead to an event of mitochondrial dysfunction and, in consequence, cytochrome *c* release [26], [27]. Therefore, we evaluated the levels of 3,3′-dihexyloxacarbocyanine iodide [DiOC_6_(3)] and 10-nonyl bromide acridine orange (NAO) fluorescence in treated cells, and it now seems likely that MJ-29 induced the level of ROS increase (Figure 2B), caused cardiolipin peroxidation (Figure 2G) and promoted the trafficking of cytochrome *c* release from mitochondria to cytosol (Figure 4A) in WEHI-3 cells. These results suggest that MJ-29-triggered apoptosis in WEHI-3 cells attributed to ROS production and the mitochondria-dependent signaling pathways.

To further demonstrate our notion that cellular ER stress responses the accumulation of unfolded or misfolded proteins triggered by chemotherapeutic agents [11], [37]. CHOP is a typical and important member of pro-apoptotic transcription factor related to ER stress [12]. The hallmarks of ER stress-regulated protein levels are up-regulated, which induced intracellular Ca^2+^ release, increased calpain level and activated caspase-12 and/or caspase-4 signals [38]. The results from our current study also clearly showed that UPR occurred in WEHI-3 cells, leading to cytosolic Ca^2+^ generation (Figure 5A) and triggering cell apoptosis. To clarify this evidence that by pre-treating with a Ca^2+^ chelator (BAPTA) in MJ-29-treated WEHI-3 cells, our results indicated the decrease of calpain 1 protein level (Figure 5E) and increased MJ-29-reduced viability (Figure 5G). Also, down-regulation of p-eIF2α expression using an ER stress inhibitor (salubrinal, which targets the eIF2α dephosphorylation) (Figure 5F) significantly blocked MJ-29-induced apoptosis in WEHI-3 cells (Figure 5G), suggesting the critical roles of eIF2α and CHOP in MJ-29-induced ER stress-mediated apoptotic death. Induction of CHOP expression is mediated through an eIF2α phosphorylation manner in MJ-29-triggered UPR (Figure 5F). Our novel findings regarding ER stress-modulated eIF2α phosphorylation and intracellular Ca^2+^ in WEHI-3 cells are critical events, leading to apoptotic cell death caused by MJ-29 treatment. Strikingly, cells were pre-incubated with *N*-acetylcysteine (NAC), an intracellular ROS scavenger, prior to MJ-29 treatment and we found that NAC significantly attenuated MJ-29-stululated the activations of CHOP and BiP protein expressions (Figure S2A) and attenuated cell death (Figure S2B) induced by MJ-29 in comparison to the treated only cells. We suggest that ROS generation plays an essential influence on MJ-29-triggered ER stress in WEHI-3 cells, and this finding is also agreement with other reports [39], [40]. Based on our functional *in vitro* study, it is demonstrated that MJ-29 not only disrupted the mitochondrial dysfunction and triggered intrinsic apoptosis but also activated the UPR in the murine myelomonocytic leukemia cell line (WEHI-3) *in vitro*.

In *in vivo* study, despite of MJ-29-chemosensitized tumor nude mice xenografts bearing subcutaneous human leukemia U937 cells [21], the present study emphasized that the establishment of an orthotopic leukemia model and elevations for the biological functions for MJ-29 regarding anti-leukemic activities and immunemodulations *in vivo*. BALB/c mice were intravenously transplanted with WEHI-3 cells, a murine monomyelocytic leukemia cell line originally derived from the BALB/c mouse [41], [42], which is an ideal system to test anti-leukemic chemotherapeutic agents [30], [31].

Consequently, we further verified striking anti-leukemic functions and found that MJ-29 inhibited WEHI-3 cells *in vivo*. As shown in Figure 6B, MJ-29 had longer the survival rate of BALB/c leukemic mice for at least 5 days by a 28-days treatment. Also, MJ-29 is able to prevent the loss of body weight when compared with the WEHI-3/leukemic mice group (Figure 6C) after 16-days exposure. The spleen or liver tissues from MJ-29-treated or untreated animals were weighed and histopathologically examined as presented in Figure 7. Our results revealed that MJ-29 significantly suppressed the enlargement of spleen (Figure 7A) and liver (Figure 7C), and the reductions of the infiltration in immature myeloblastic cells into splenic red pulp (Figure 7E) were also observed in MJ-29-treated leukemic mice. It is reported that immature myeloblastic cells entering the leukocytes can be discovered in case of the leukemic cells metastasized to the liver tissues [43]. Neoplastic cells are not easy to discover in the red pulp of the spleens from chemotherapeutic leukemic mice [30]. It is noteworthy that regarding the side effects and toxicity of MJ-29 on normal and leukemic mice, and no significant difference in blood chemistry values within treated animals was found compared to both doses of MJ-29 and control groups (Table S1 and Method S2). Moreover, there are significant differences in the increases in the amount of T and B cells (CD3 and CD19, respectively) rather than the decreases of monocytes and macrophages (CD11b and Mac-3, respectively) between the leukemic mice treated or un-treated groups (Figure 6D and Figure S3). These results might be involved in the reason why WEHI-3 cells, originally designated as a myelomonocytic cell line [41], stimulated that of T and B cells but decreased in the levels of macrophages and monocytes from MJ-29-treated leukemic mice. Also, we proposed that MJ-29 modulated immune responses through not only increasing T- and B-cells proliferations and phagocytotic activity by macrophages but also promoting NK cell cytotoxicity in leukemic mice *in vivo* (Figure 8). It is well known that that macrophages are major roles to innate immunity [44], [45], and stimulation of NK cell cytotoxicity could contribute to the increase immune response [46]. Thus, we suggest that MJ-29 might possess anti-leukemic activity partially through modulating immune responses in BALB/c mice. However, MJ-29 if directivity affects immunemodulations to reach anti-leukemia is indeed for further investigations.

In conclusions, our study is the first report to provide an approach regarding that the newly quinazolinone MJ-29 tends to inhibit murine myelomonocytic leukemia WEHI-3 cells *in vitro* and *in vivo*. The induction of cell death in MJ-29-treated WEHI-3 cells has therefore been proven by an assessment of apoptosis *in vitro* culture through not only intrinsic apoptotic pathway but also ER stress signaling. Strikingly, the orthotopic leukemic mice were exposed to MJ-29 in *in vivo* model, and it is shown that anti-leukemic responses occurred when the increased immune response *in vivo*. For that reason, we presented our novel findings and the efficacy of MJ-29 might be sufficient to investigate the potential of leukemia treatment in the future.

## Materials and Methods

### Cell culture

The murine myelomonocytic leukemia cell line (WEHI-3) was purchased from the Bioresource Collection and Research Center (BCRC), Food Industry Research and Development Institute (FIRDI) (Hsinchu, Taiwan). Cells plated in 75-cm^2^ cell culture flasks were grown in RPMI 1640 medium with supplements plus 10% (v/v) fetal bovine serum, 2 mM L-glutamine, 100 Units/ml penicillin and 100 µg/ml streptomycin at 37°C under a humidified 5% (v/v) CO_2_ one atmosphere in an incubator. Cells were split and centrifuged every two days to maintain cell growth before experiments [31], [47].

### Chemicals and reagents

DMSO, PI, RNase A and Triton X-100 were obtained from Sigma-Aldrich Corp. (St. Louis, MO, USA). Cell culture materials were purchased from Gibco/Life Technologies (Carlsbad, CA, USA). DAPI, 2′,7′-dichlorodihydrofluorescein diacetate (H_2_DCFDA), DiOC_6_(3), MitoProbe Transition Pore Assay kit, NAO, Fluo-3/AM and BAPTA were purchased from Molecular Probes/Life Technologies (Eugene, OR, USA). Caspase-9 and caspase-3 substrate reagent kits (CaspaLux-9 M_1_D_2_ and PhiPhiLux G_1_D_2_) were bought from OncoImmunin, Inc. (Gaithersburg, MD, USA). The specific caspase inhibitors (Z-LEHD-FMK for caspase-9 and Z-DEVD-FMK for caspase-3) were purchased from BioVision, Inc. (Mountain View, CA, USA). Anti-Bax, anti-Bcl-2, anti-cleaved caspase-3, anti-cleaved caspase-9, anti-eIF2α and anti-phospho-eIF2α (Ser51) were obtained from Cell Signaling Technology (Beverly, MA, USA). The other primary antibodies used in this study and salubrinal were bought from Santa Cruz Biotechnology, Inc. (Santa Cruz, CA, USA). MJ-29 as shown in the top of Figure 1A was synthesized and provided by Mann-Jen Hour, Ph.D. (School of Pharmacy, College of Pharmacy, China Medical University).

### Determination of cell viability and morphological changes

Cells at a density of 2×10^5^ cells/well were placed in 24-well plates and treated with DMSO alone [0.1% (v/v) in media served as a vehicle control] and different concentrations (0.5, 1, 5 or 10 µM) of MJ-29 for 24 h. For measuring the viability, cells from each sample were collected and labeled with PI (4 µg/ml). Live and dead cells were determined by a PI exclusion method as previously described [21], [26]. Cells were immediately analyzed by a Becton Dickinson FACSCalibur flow cytometer (BD Biosciences, Franklin Lakes, NJ, USA) and calculated utilizing BD CellQuest Pro software. After incubation, treated cells were photographed under a phase-contrast microscope before being harvested [26].

### Analysis for DNA content and sub-G1 populations by flow cytometry

Approximately 2×10^5^ cells per well were seeded in 24-well plates and then exposed to 1 µM of MJ-29 for 12, 18 and 24 h. The trypsinized cells were washed with PBS and fixed with ice-cold 70% (v/v) ethanol at −20°C overnight. After being washed, cells were stained with PI at a concentration of 40 µg/ml in the presence of 0.1% (v/v) Triton X-100 and RNase A (20 µg/ml) for 30 min in a dark room. The apoptotic cells were quantified by measuring the sub-G1 DNA content using the PI method. Each sample was analyzed, and fluorescence intensity of DNA content in the FL-2 channel was determined and monitored by flow cytometry as described elsewhere [48].

### Assay for apoptosis by annexin V/PI double staining

Cells (2×10^5^ cells/well) after exposure to 1 µM of MJ-29 for 0, 12 and 24 h were trypsinized and harvested before incubation with annexin V and PI. Apoptotic cells were determined utilizing an Annexin V-FITC Apoptosis Detection kit (BD Biosciences Pharmingen, San Diego, CA, USA) according to the manufacturer's protocol. Ten thousand cells were measured per sample by flow cytometry, and the analysis of apoptotic cells was performed using BD CellQuest Pro software as previously described [35], [49].

### Nuclear staining with DAPI for apoptosis

Cells (1×10^5^ cells per well) cultured in 24-well plates were treated with or without 1 µM of MJ-29 following a 24-h treatment. Cells were washed with PBS, fixed in 4% (v/v) formaldehyde (Sigma-Aldrich Corp.) for 15 min and permeabilized by sequentially treating with 0.1% (v/v) Triton X-100 for another 15-min exposure. A 200-µl DAPI solution (1 µg/ml) was added into each well for 30 min at 37°C in the dark and thereafter visualized using a fluorescence microscope (Nikon Inc., Tokyo, Japan) [35], [50]. Quantification of apoptotic cells was performed utilizing Metamorph Imaging System (Universal Imaging Corp., Downingtown, PA, USA) in three random fields from each well.

### TUNEL assay by flow cytometry

TUNEL assay was used to determine apoptotic DNA breaks utilizing the *in situ* Cell Death Detection Kit, Fluorescein (Roche Diagnostics, Boehringer Mannheim, Mannheim, Germany). Briefly, cells at a density of 2×10^5^ cells/well in 24-well plates were treated with 1 µM of MJ-29 for 0, 6, 12 and 24 h. After the end of treatment, cells were harvested and followed the protocol provided by the manufacturer. TUNEL positive cells were measured by flow cytometry as previously described [30], [51].

### Determinations of ROS productions, ΔΨm, MPT pores and level of cardiolipin oxidation by flow cytometry

Cells at an initial density of 2×10^5^ cell/ml in 24-well plates were incubated with 1 µM of MJ-29 for 3, 6, 12 and 24 h or vehicle control to detect if this compound would affect ROS, ΔΨm, the opening of MPT pores and cardiolipin oxidation which were measured as previously described [35], [50], [52]. Cells were harvested, washed with PBS twice and re-suspended in 500 µl of H_2_DCFDA (10 µM) for ROS, DiOC_6_(3) (500 nM) for ΔΨm and NAO (500 nM) for cardiolipin oxidation at 37°C for an additional 30 min. The opening of MPT pores in MJ-29-treated WEHI-3 cells was monitored using the MitoProbe Transition Pore Assay kit (Molecular Probes/Life Technologies) and performed according procedures provided by the manufacturer [35], [53].

### Flow cytometric analysis for caspase-9 and caspase-3 activities and intracellular Ca^2+^ level

Cells (2×10^5^ cells/well) in 24-well plates were treated with MJ-29 (1 µM) for 6, 12 and 24-h treatments or vehicle only. Cells were then harvested from each treatment, and activities of caspase-9 (CaspaLux-9 M_1_D_2_ kit) and caspase-3 (PhiPhiLux G_1_D_2_ kit) were determined according to the manufacturer's protocol (OncoImmunin, Inc., Gaithersburg, MD, USA) as previously described [48]. For determining intracellular Ca^2+^ level, cells were stained with Ca^2+^ indicator Fluo-3/AM (2.5 µg/ml) at 37°C for 40 min after 1 µM of MJ-29 exposure for 3, 6, 12 and 24 h. Flow cytometric analysis was used to detect the influences on the level of intracellular Ca^2+^ as described elsewhere [35].

### Western blotting analysis for protein levels

Cells in 6-well plates at a density of 1×10^6^ cells/well were treated in the presence or absence of MJ-29 (1 µM) for 6, 12 and 24 h. At the end of each incubation, cells were harvested, washed twice with cold PBS and centrifuged at 1,000×g for 5 min. Cell protein was extracted into the PRO-PREP protein extraction solution (iNtRON Biotechnology, Seongnam-si, Gyeonggi-do, Korea), and protein concentration was determined by the Bio-Rad protein assay (Bio-Rad Laboratories, Hercules, CA, USA). Protein samples (30 µg) were loaded on a 10% (w/v) polyacrylamide gel and subjected to sodium dodecyl sulfate-polyacrylamide gel electrophoresis (SDS-PAGE) [35], [48]. Samples were then transferred to Immobilon-P transfer membrane (Millipore, Bedford, MA, USA) and the membranes blocked in PBST [1× PBS and 0.1% (v/v) Tween 20 containing 5% (w/v) nonfat dry milk for 2 h at room temperature, and hybridized with appropriate primary antibodies (1∶1000 dilution) overnight at 4°C followed by horseradish peroxidase (HRP)-conjugated secondary antibodies. Blots were detected using the enhanced chemiluminescence (ECL) western blotting detection kit (Immobilon Western HRP substrate, Millipore) and autoradiography utilizing X-ray film (GE Healthcare, Piscataway, NJ, USA) [26], [35]. The band densities were quantified using the NIH ImageJ 1.45 program (Bethesda, MI, USA). The blots were reprobed with an anti-β-Actin antibody (Sigma-Aldrich Corp.) for equal protein loading.

### Effects of inhibitors of apoptosis and ER stress on cytotoxicity and abundance of protein levels induced by MJ-29

Cells were pretreated with or without 10 µM of specific caspase inhibitors (Z-LEHD-FMK for caspase-9 and Z-DEVD-FMK for caspase-3, respectively) for 2 h before the finish of MJ-29 treatment (1 µM) for 24 h, and cell viability was determined using a PI exclusion method as described above. For inhibition of ER stress-mediated apoptosis, both of Ca^2+^ chelator (BAPTA) and ER stress inhibitor (salubrinal, an eIF2α dephosphorylation inhibitor) were applied. Cells were pre-incubated with the BAPTA (5 µM) or salubrinal (10 µM) for 2 h and thereafter exposed to 1 µM of MJ-29. After a 24-h treatment, cells were harvested for measuring viability using flow cytometric analysis and determining ER stress-related protein levels by immunoblotting as previously described [26], [35].

### Immunofluorescence and co-localization of proteins by confocal microscopy

Cells plated on 4-well chamber slides at a density 5×10^4^ cells/well were treated with 1 µM of MJ-29 for 24 h. Cells were then fixed in 3% (v/v) formaldehyde (Sigma-Aldrich Corp.) in PBS for 15 min, permeabilized in 0.1% (v/v) Triton X-100 in PBS for 30 min and stained with the primary antibodies (anti-cytochrome *c* and anti-CHOP/GADD153 diluted at 1∶200) at 4°C overnight, followed utilizing FITC-conjugated secondary antibodies (green fluorescence, Invitrogen/Life Technologies) at 1∶100 dilutions for 1 h at room temperature. Cells were washed twice with PBS and individually stained with CellTracker Red CMTPX (Molecular Probes/Life Technologies) for cytosol and with PI for nucleus (red fluorescence). Coverslips were mounted, and photomicrographs were obtained using a Leica TCS SP2 confocal spectral microscope (Leica Microsystems, Heidelberg, Mannheim, Germany) as previously described [26], [35].

### Animals' administration

Eighty five-week-old male BALB/c mice (approximately 20–25 g body weight) were obtained from the National Laboratory Animal Center (Taipei, Taiwan). Mice were housed in a regular 12-h light/12-h dark cycle, bred with clean water and fed commercial diet *ad libitum* in standard conditions of constant temperature and humidity. The start of our experiment worked after keeping at least one week. All animal studies were conducted according to institutional guidelines (Affidavit of Approval of Animal Use Protocol, No. 97-33-N) approved by the Institutional Animal Care and Use Committee (IACUC) of China Medical University (Taichung, Taiwan).

### Establishment of the orthotopic leukemic mice and MJ-29 treatment

All mice were randomly divided into 8 groups, and each group contains 10 animals. Group I and V are a normal control group; and group II and VI were intravenously injected with WEHI-3 cells (1×10^6^ cells in 100 µl PBS per mouse) only as a model of leukemic mice. Groups III and IV as well as VII and VIII were intravenously injected with WEHI-3 cells (1×10^6^ cells/mouse) for a 7-day incubation periods, and the animals were then treated with MJ-29 (10 and 20 mg/kg body weight, respectively) by intraperitoneal administration once every two days [21].

### The *in vivo* studies are divided into two parts

#### Part I. survival analysis for MJ-29-treated orthotopic leukemic mice

The animals in groups V, VI, VII and VIII were treated with or without MJ-29 at 10 and 20 mg/kg. These groups were administrated for 21–28 days for measuring the survival rate. The Kaplan-Meier estimator for the survival function was expressed as previously described [47], [54], [55]. Anti-leukemic activity was performed as the ratio of median survival time (MST) of treated or untreated leukemic mice (T) groups versus that of the control (C) group. Data for survival rate (%) was undertaken as the following formula: life span T/C (%) = (MST of T group/MST of C group)×100.

#### Part II. Evaluations for anti-leukemic activity

MJ-29 was administered for every other day up to 16 days by intraperitoneal injection at 10 and 20 mg/kg in leukemic mice. At the end of the experiment, all animals were sacrificed by euthanasia with carbon dioxide (CO_2_) followed by cervical dislocation [31].

### Assessments for the weights of body, spleen and liver tissues

Body weight of each mouse during treatment was recorded once every four days for 16 days. After finishing treatment, each animal was sacrificed on day 16 before blood was collected, and spleen and liver samples were obtained and weighed individually as previously described [31], [47].

### Histopathology of spleen tissue by hematoxylin-eosin stain

The spleen tissues from leukemic mice were isolated and subjected to histopathological analysis. Spleen was fixed in 4% (v/v) formaldehyde, embedded in paraffin, sectioned at 5 µm, and stained with hematoxylin and eosin according to the previous procedure [31].

### Blood samples collection and immunofluorescence staining

About 500 µl blood was collected from each mouse in different groups and then immediately exposed to 1× Pharm Lyse lysing buffer (BD Biosciences, San Jose, CA, USA) for lysing of the red blood cells followed by centrifugation for 5 min at 1500 rpm at 4°C. The isolated leukocytes were examined for cell markers based on being stained with FITC-conjugated anti-mouse CD3, PE-conjugated anti-mouse CD19, PE-conjugated anti-mouse Mac-3 and FITC-conjugated anti-mouse CD11b antibodies (BD PharMingen Inc, San Diego, CA, USA). Subsequently, cells were determined for the levels of specific cell surface markers by flow cytometry as described elsewhere [31], [47].

### Detection for phagocytic activity by macrophages

The heparinized blood samples or peritoneal macrophages from each mouse in MJ-29-treated or un-treated groups were isolated as previously described [56]. Approximately 1×10^5^ leukocytes in 100 µl of samples were incubated for 1 h at 37°C with FITC-labeled opsonized *Escherichia coli* (*E. coli*) (at a density of 2×10^7^ bacteria per 20 µl 1× solution from the PHAGOTEST kit, Glycotope Biotechnology GmbH, Czernyring, Heidelberg, Germany). The reaction was stopped by the addition of quenching solution (100 µl) according to the manufacturer's instruction, and the whole blood is then lysed with 2 ml of 1× lysing solution for 20 min at room temperature. After the completion of phagocytosis, DNA was stained and cells from each sample were analyzed by flow cytometery as previously described [47], [57]. Fluorescent particles were collected on 10,000 cells and data were analyzed using the BD CellQuest Pro software.

### Assay for NK cell cytotoxicity

The fresh spleens from all experimental mice were processed to isolate splenocytes, and about 1×10^5^ splenocytes were cultured in each well of 24-well culture plates. YAC-1 cells obtained from the BCRC (Hsinchu, Taiwan) were stained according to the manufacturer's protocols (PKH67 Fluorescent Cell Linker Kits, Sigma-Aldrich Corp.) [47], [58]. The labeled YAC-1 cells (about 1×10^6^ cells per 100 µl) were placed on 96-well plates before the addition of the splenocytes from each treatment to the wells after a 12-h incubation and determination of NK cell cytotoxicity by a PI exclusion assay and flow cytometry as previously described [47], [58].

### Assessments of T- and B-cell proliferation

Splenocytes (1×10^5^ cells/well) from each mouse seeded in 100 µl of RPMI 1640 medium with 10% (v/v) fetal bovine serum in 96-well plates were stimulated with Con A (1 µg/ml; Sigma-Aldrich Corp.) for T-cell and LPS (1 µg/ml; Sigma-Aldrich Corp.) for B-cell for 3 and 5 days incubations, respectively. T- and B-cell proliferation was determined by the CellTiter 96 AQueous One Solution Cell Proliferation Assay kit (Promega, Madison, WI, USA) as previously described [47], [58].

### Statistical analysis

Data are represented as means ± standard deviation (S.D.) from at least three independent experiments. The values are analyzed by one-way analysis of variance (ANOVA) followed by Tukey's HSD test. Cases in which *p*-value of less than 0.05 was accepted to have a pronounced statistically difference between experimental and control samples. In *in vivo* experiment, survival of the mice was measured from the date of pair matching to sacrifice (event) or end of study (censored). The Kaplan-Meier method was used to estimate survival when comparing survival between leukemic mice and the respective treatment groups.

## Supporting Information

Methods S1 **Expanded description of evaluation protocols for Figure S1A and Table S1.**
(PDF)

Figure S1 **MJ-29 reduces cell viability and triggers apoptotic death in WEHI-3 cells.** Cells were treated with different concentrations (0.5, 1, 5 or 10 µM) of MJ-29 for 24 h or 1 µM of MJ-29 for 12 and 24 h. (A) MJ-29 concentration-dependently inhibited the cell viability, which was determined by MTT assay and the percent viabilities were plotted as the means ± S.D. of at least three experiments. *p<0.05 compared with 0.1% (v/v) DMSO-treated vehicle-treated control cells by Tukey's HSD test. (B) The representative profiles from BD CellQuest Pro software indicated that DNA content for distribution of cell cycle by PI-stained assay and (C) apoptotic cells (annexin V-FITC positive) by annexin V/PI staining in the presence of 1 µM of MJ-29 for 12 and 24 h were determined utilizing flow cytometry. Quantifications of annexin V positive cells were measured as described in the “Materials and Methods”. The data conducted three times with similar results.
(TIFF)

Figure S2 **ROS and ER-stress-mediated apoptosis occurs in MJ-29-treated WEHI cells.** Cells were pretreated with or without 10 mM of NAC (Sigma-Aldrich Corp.), a ROS scavenger for 1 h and then exposed to 1 µM of MJ-29 for 24 h. At the end of treatment, cells were collected and determined the hallmark protein levels of ER stress and viability in MJ-29-treated cells as described in the “Materials and Methods”. (A) The protein expressions of CHOP and BiP were performed by Western blotting. β-Actin was an internal control. Results shown are representative of three independent experiments. (B) Abrogation of MJ-29-reduced cell viability by NAC was detected by flow cytometric analysis and analyzed utilizing BD CellQuest Pro software. Results are shown as means ± S.D. in triplicate and determined by Tukey's HSD test. *, *p*<0.05, shows significant difference compared with 0.1% (v/v) DMSO vehicle control; #, *p*<0.05, is significantly different compared to only MJ-29-treated cells.
(TIFF)

Figure S3 **MJ-29 alters the levels of CD surface markers in leukemic mice.** Animals were intravenously injected with WEHI-3 cells (1×10^6^ cells/100 µl) and intraperitoneally treated with MJ-29 (10 and 20 mg/kg) every other day for 16 days. Whole blood was collected from individual mice, and leukocytes were analyzed the with specific cell surface markers by flow cytometry. (A) The profiles of anti-CD3-PE for T lymphocytes and anti-CD19-FITC for B cells from BD CellQuest Pro software were shown, and (B) that of anti-Mac-3-PE for macrophages and anti-CD11b-FITC for monocytes were revealed as described in the “Materials and Methods”.
(TIFF)

Table S1 **Blood biochemical profiles in the BALB/c mice with or without WEHI-3 cells by intravein transplantation following treatment of MJ-29 by intraperitoneal injection.**
(PDF)
