# Supplementary material for: Novel Quinazolinone MJ-29 Triggers Endoplasmic Reticulum Stress and Intrinsic Apoptosis in Murine Leukemia WEHI-3 Cells and Inhibits Leukemic Mice
Source: PLoS One. 2012 May 25;7(5):e36831. doi: 10.1371/journal.pone.0036831 (PMC3360742; doi:10.1371/journal.pone.0036831)
Supplement: Methods S1 — Expanded description of evaluation protocols for Figure S1A and Table S1. (PDF) [file pone.0036831.s001.pdf]

Supporting Information

Methods S1

3-(4,5-dimethylthiazol-2-yl)-2,5-diphenyltetrazolium bromide (MTT) assay

The effects of MJ-29 on cell metabolic status in WEHI-3 were assessed using the reduction of MTT to colored blue formazan product [1,2]. In brief, cells (1×10^4 cells/well) into 96-well plates were incubated in the absence [0.1% (v/v) DMSO vehicle control] or presence of the various concentrations (0.5, 1, 5 or 10 μM) of MJ-29 for 24 h. After exposure, a 10-μl of MTT (5 mg/ml, Sigma-Aldrich Corp.) was individually added into each well for 3 h at 37°C. The formazan crystals were solubilized by adding 100 μl of 10% (w/v) sodium dodecyl sulfate (SDS, Sigma-Aldrich Corp.) in 0.1 N HCl per well and subsequently the colored solution was measured by absorbance at 570 nm with a microplate reader as described previously [3]. The optical density (OD) of blank wells containing medium and MTT, but no cells, was subtracted, and results are expressed as % of MTT reduction compared to untreated control conditions.

Levels of biochemical measurements

Mice were monitored the drug-related toxicity of each group after the animals were sacrificed, and whole blood were harvested for biochemical measurements for evaluating for the safety of MJ-29. In brief, blood was collected from each mouse, allowed to clot and centrifuged at 1000 ×g for 10 min at room temperature to determine these biochemical tests as previously elsewhere [4,5,6].

References

1. Mosmann T (1983) Rapid colorimetric assay for cellular growth and survival: application to proliferation and cytotoxicity assays. J Immunol Methods 65:

55-63.

2. Dejeans N, Tajeddine N, Beck R, Verrax J, Taper H, et al. (2010) Endoplasmic reticulum calcium release potentiates the ER stress and cell death caused by an oxidative stress in MCF-7 cells. Biochem Pharmacol 79: 1221-1230.
3. Chang YH, Yang JS, Kuo SC, Chung JG (2009) Induction of mitotic arrest and apoptosis by a novel synthetic quinolone analogue, CWC-8, via intrinsic and extrinsic apoptotic pathways in human osteogenic sarcoma U-2 OS cells. Anticancer Res 29: 3139-3148.
4. Xing X, Liu V, Xia W, Stephens LC, Huang L, et al. (1997) Safety studies of the intraperitoneal injection of E1A--liposome complex in mice. Gene Ther 4: 238-243.
5. Hsu SC, Ou CC, Li JW, Chuang TC, Kuo HP, et al. (2008) Ganoderma tsugae extracts inhibit colorectal cancer cell growth via G(2)/M cell cycle arrest. J Ethnopharmacol 120: 394-401.
6. Chiang JH, Yang JS, Ma CY, Yang MD, Huang HY, et al. (2011) Danthron, an anthraquinone derivative, induces DNA damage and caspase cascades-mediated apoptosis in SNU-1 human gastric cancer cells through mitochondrial permeability transition pores and Bax-triggered pathways. Chem Res Toxicol 24: 20-29.
