# Supplementary figures and images for: Novel Quinazolinone MJ-29 Triggers Endoplasmic Reticulum Stress and Intrinsic Apoptosis in Murine Leukemia WEHI-3 Cells and Inhibits Leukemic Mice
Source: PLoS One. 2012 May 25;7(5):e36831. doi: 10.1371/journal.pone.0036831 (PMC3360742; doi:10.1371/journal.pone.0036831)

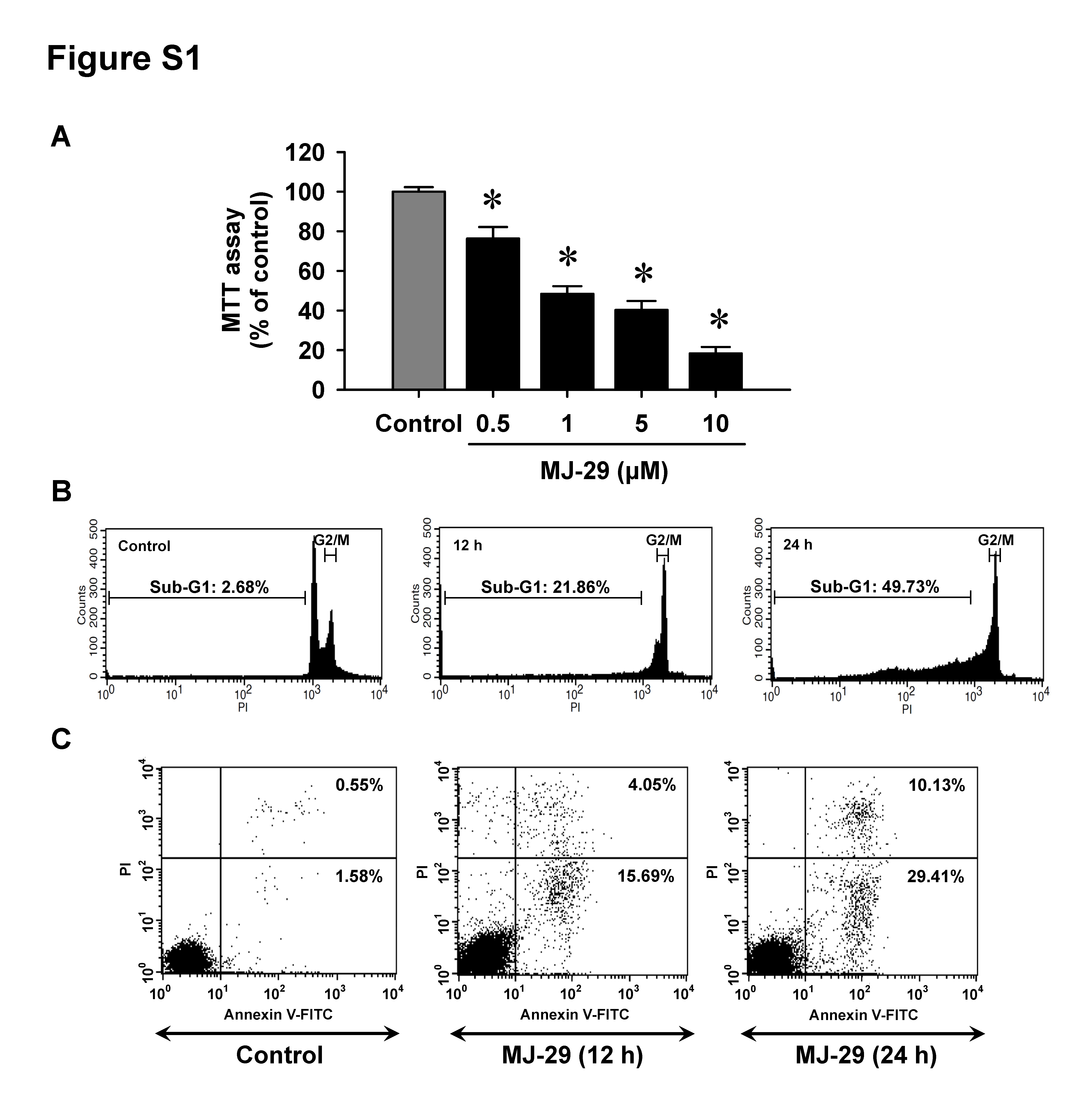

Supplement: Figure S1 — MJ-29 reduces cell viability and triggers apoptotic death in WEHI-3 cells. Cells were treated with different concentrations (0.5, 1, 5 or 10 µM) of MJ-29 for 24 h or 1 µM of MJ-29 for 12 and 24 h. (A) MJ-29 concentration-dependently inhibited the cell viability, which was determined by MTT assay and the percent viabilities were plotted as the means ± S.D. of at least three experiments. *p<0.05 compared with 0.1% (v/v) DMSO-treated vehicle-treated control cells by Tukey's HSD test. (B) The representative profiles from BD CellQuest Pro software indicated that DNA content for distribution of cell cycle by PI-stained assay and (C) apoptotic cells (annexin V-FITC positive) by annexin V/PI staining in the presence of 1 µM of MJ-29 for 12 and 24 h were determined utilizing flow cytometry. Quantifications of annexin V positive cells were measured as described in the “Materials and Methods”. The data conducted three times with similar results. (TIFF) [file pone.0036831.s002.tiff]

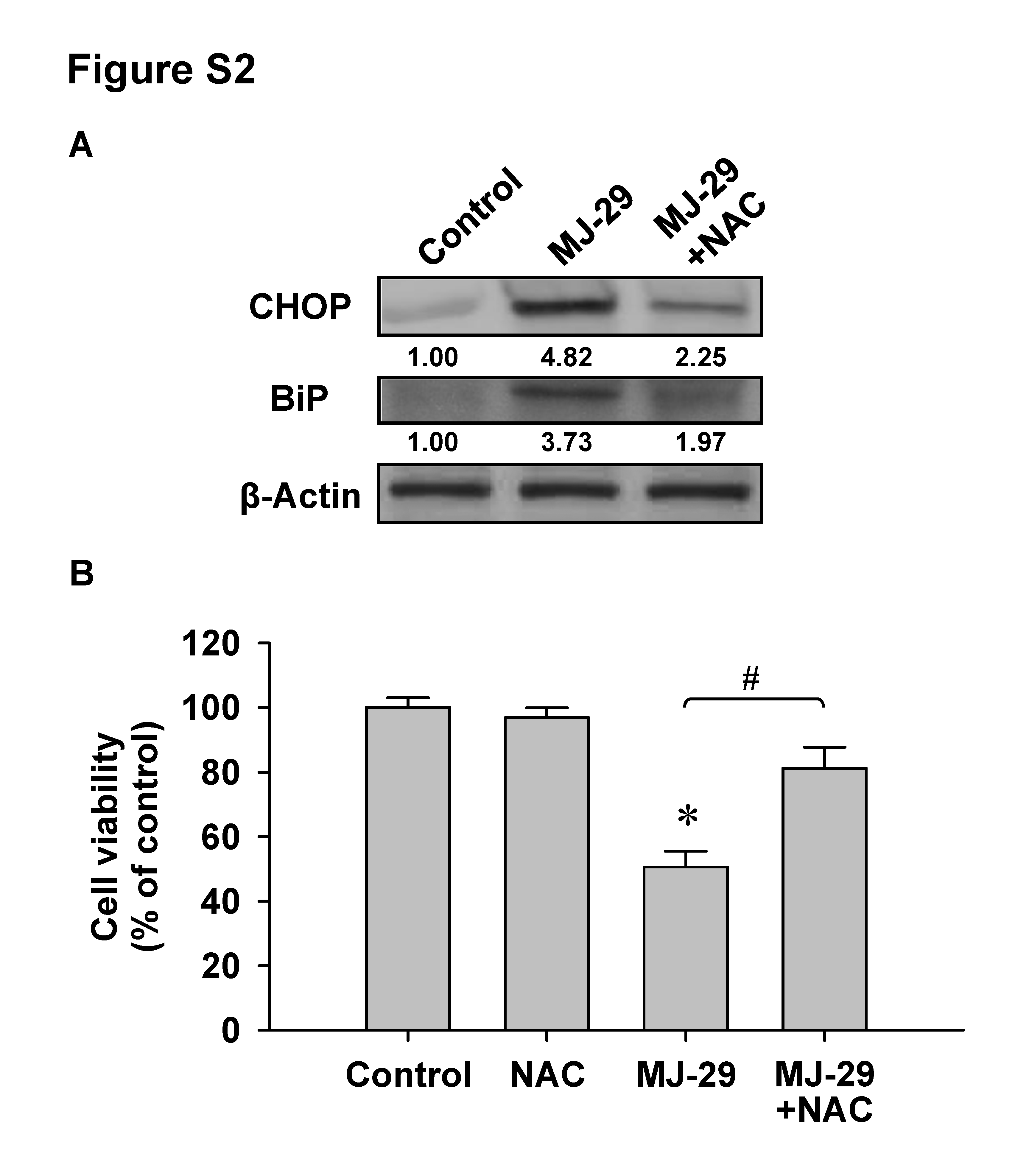

Supplement: Figure S2 — ROS and ER-stress-mediated apoptosis occurs in MJ-29-treated WEHI cells. Cells were pretreated with or without 10 mM of NAC (Sigma-Aldrich Corp.), a ROS scavenger for 1 h and then exposed to 1 µM of MJ-29 for 24 h. At the end of treatment, cells were collected and determined the hallmark protein levels of ER stress and viability in MJ-29-treated cells as described in the “Materials and Methods”. (A) The protein expressions of CHOP and BiP were performed by Western blotting. β-Actin was an internal control. Results shown are representative of three independent experiments. (B) Abrogation of MJ-29-reduced cell viability by NAC was detected by flow cytometric analysis and analyzed utilizing BD CellQuest Pro software. Results are shown as means ± S.D. in triplicate and determined by Tukey's HSD test. *, p<0.05, shows significant difference compared with 0.1% (v/v) DMSO vehicle control; #, p<0.05, is significantly different compared to only MJ-29-treated cells. (TIFF) [file pone.0036831.s003.tiff]

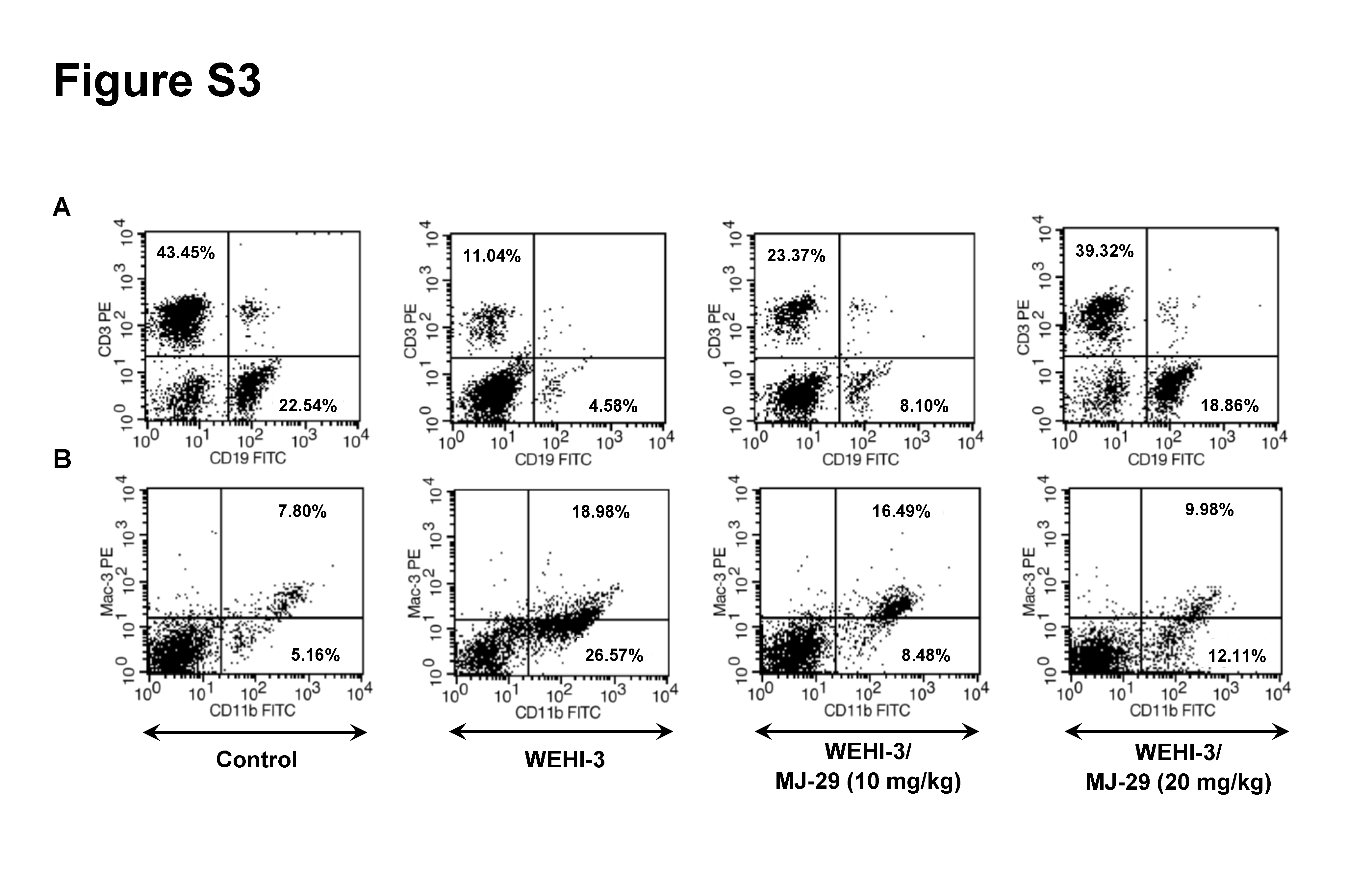

Supplement: Figure S3 — MJ-29 alters the levels of CD surface markers in leukemic mice. Animals were intravenously injected with WEHI-3 cells (1×10^6^ cells/100 µl) and intraperitoneally treated with MJ-29 (10 and 20 mg/kg) every other day for 16 days. Whole blood was collected from individual mice, and leukocytes were analyzed the with specific cell surface markers by flow cytometry. (A) The profiles of anti-CD3-PE for T lymphocytes and anti-CD19-FITC for B cells from BD CellQuest Pro software were shown, and (B) that of anti-Mac-3-PE for macrophages and anti-CD11b-FITC for monocytes were revealed as described in the “Materials and Methods”. (TIFF) [file pone.0036831.s004.tiff]
