# Supplementary material for: Novel Quinazolinone MJ-29 Triggers Endoplasmic Reticulum Stress and Intrinsic Apoptosis in Murine Leukemia WEHI-3 Cells and Inhibits Leukemic Mice
Source: PLoS One. 2012 May 25;7(5):e36831. doi: 10.1371/journal.pone.0036831 (PMC3360742; doi:10.1371/journal.pone.0036831)
Supplement: Table S1 — Blood biochemical profiles in the BALB/c mice with or without WEHI-3 cells by intravein transplantation following treatment of MJ-29 by intraperitoneal injection. (PDF) [file pone.0036831.s005.pdf]

Table S1. Blood biochemical profiles in the BALB/c mice with or without WEHI-3 cells by intravein transplantation following treatment of MJ-29 by intraperitoneal injection

			**WEHI-3/MJ-29**	
**Item[a,b]**	**Control**	**WEHI-3**	**10 mg/kg**	**20 mg/kg**
ALB (g/dL)	2.95±0.07	2.50±0.03	2.60±0.04	2.50±0.06
ALT (U/L)	110.00±5.66	126.00±17.79	81.00±14.91	100.00±16.45
AST (U/L)	215.50±14.85	249.00±12.51	200.70±17.78	230.00±21.45
BUN (mg/dL)	21.35±0.92	21.30±1.02	19.40±1.78	22.50±1.27
CREA (mg/dL)	0.48±0.04	0.59±0.10	0.46±0.09	0.37±0.17
GLU (mg/dL)	177.50±6.36	162.00±7.68	158.70±8.87	166.58±6.36
LDH (U/L)	296.00±26.87	356.80±62.45	259.00±31.13	295.30±45.81
TP (g/dL)	4.95±0.21	4.40±0.17	4.20±0.14	4.20±0.20
UA (mg/dL)	2.05±0.07	1.70±0.09	3.70±0.11	3.60±0.12

[a]ALB, albumin; ALT, alanine transaminase; AST, aspartate transaminase; BUN, blood urea nitrogen; CREA, creatinine; GLU, glucose; LDH, lactate dehydrogenase; TP, total protein; UA, uric acid

[b]Values represent as mean ± S.D. from at least five samples per group
